# Benchmark assessment of molecular geometries and energies from small molecule force fields

**DOI:** 10.12688/f1000research.27141.1

**Published:** 2020-12-03

**Authors:** Victoria T. Lim, David F. Hahn, Gary Tresadern, Christopher I. Bayly, David L. Mobley

**Affiliations:** 1 Department of Chemistry, University of California, Irvine, CA, 92697, USA; 2 Computational Chemistry, Janssen Research & Development, Beerse, B-2340, Belgium; 3 OpenEye Scientific Software, Santa Fe, NM, 87507, USA; 4 Department of Pharmaceutical Sciences, University of California, Irvine, CA, 92697, USA

**Keywords:** force field, molecular modeling, OpenFF, OPLS, molecular mechanics, molecular dynamics, quantum mechanics

## Abstract

**Background:** Force fields are used in a wide variety of contexts for classical molecular simulation, including studies on protein-ligand binding, membrane permeation, and thermophysical property prediction. The quality of these studies relies on the quality of the force fields used to represent the systems.

**Methods:** Focusing on small molecules of fewer than 50 heavy atoms, our aim in this work is to compare nine force fields: GAFF, GAFF2, MMFF94, MMFF94S, OPLS3e, SMIRNOFF99Frosst, and the Open Force Field Parsley, versions 1.0, 1.1, and 1.2. On a dataset comprising 22,675 molecular structures of 3,271 molecules, we analyzed force field-optimized geometries and conformer energies compared to reference quantum mechanical (QM) data.

**Results:** We show that while OPLS3e performs best, the latest Open Force Field Parsley release is approaching a comparable level of accuracy in reproducing QM geometries and energetics for this set of molecules. Meanwhile, the performance of established force fields such as MMFF94S and GAFF2 is generally somewhat worse. We also find that the series of recent Open Force Field versions provide significant increases in accuracy.

**Conclusions:** This study provides an extensive test of the performance of different molecular mechanics force fields on a diverse molecule set, and highlights two (OPLS3e and OpenFF 1.2) that perform better than the others tested on the present comparison. Our molecule set and results are available for other researchers to use in testing.

## Introduction

The study of chemical and biological systems relies on an accurate assessment of the energetics and geometries of the systems. Many computational methods serve to help investigate these systems, ranging from more accurate, higher cost quantum mechanical techniques to more approximate methods which compromise accuracy in favor of increased eﬃciency. Classical mechanics-based calculations fall into the latter group, and have an advantage over more theoretically rigorous calculations of being able to model larger systems over longer time timescales ^1– 4^.

The modeling and simulation of molecular systems in classical mechanical calculations typically requires a force field, a set of energy functions and associated parameters comprising the potential energy function. This potential energy function defines interactions between components in the system based on the coordinates of its particles ^5, 6^.

Force fields have a long history of development. Strategies for force field development vary in terms of the chemical space covered, the types of data used for training, and the approach to optimize parameters given a set of input data ^7– 10^. The training data used to develop a force field usually includes input data from both experimental and reference quantum mechanical (QM) calculations. This finite amount of input data is carefully chosen to be representative of the systems for which the force field is designed. The limit of accuracy for some force field is measured by its ability to reproduce experimental observables, such as hydration free energies. When experimental evidence is unavailable, the force field can be assessed with respect to quantum mechanical data, for instance its ability to reproduce QM geometries and relative energies. Given the complexity of force field development, including multidimensional input data, various functional forms, and approaches to chemical perception ^11^, force fields vary in how accurately they can compute properties of interest. Indeed, many examples serve to highlight the limitations of force fields ^12– 16^.

Our focus in this work is on force fields for small molecules, which are instrumental in drug discovery; for instance, evaluating binding free energies and modeling ligand binding poses. Relatively few literature studies evaluate force field accuracy on general small drug-like molecules, in contrast to force fields for proteins ^17– 25^, nucleic acids ^26– 28^, carbohydrates ^29– 32^, and other specific chemical systems ^33– 45^. On small molecules, these studies comprise predictions of solvation free energies ^46, 47^, strain energies ^48^ experimental osmotic coeﬃcients ^49^, partition coeﬃcients ^50, 51^, conformer energies ^52– 56^, conformer geometries ^55, 57^, and robustness of parameterization ^57^. Most of these studies assess four or fewer force fields on molecule sets up to several hundreds of molecules. We present a broader assessment of general small molecule force fields on a large, diverse library of drug-like compounds and evaluate how accurately these force fields perform. We use QM data as a valuable source of information for force field assessment and to explore chemical space relatively quickly and easily.

In this work, we benchmarked small molecule force fields with respect to quantum mechanical results. We assessed nine force fields belonging to four families: the General Amber Force Field, first and second generations (GAFF ^58^ and GAFF2 ^59^); the Merck Molecular Force Field, initial and “static” versions (MMFF94 ^60– 64^ and MMFF4S ^56, 65^); the third extended version of the Optimized Potentials for Liquid Simulations Force Field (OPLS3e ^66^); and the SMIRKS-based force fields from the Open Force Field Initiative (SMIRNOFF99Frosst ^67^ and its successor OpenFF “Parsley” ^68^, versions 1.0, 1.1, and 1.2). For a dataset of 22,675 molecular structures of 3,271 small molecules, we conducted molecular mechanics (MM) energy minimizations using force fields and evaluated optimized geometries and energies, compared with reference to quantum mechanical data. We also identified particular chemical groups that represent systematic outliers in the force field-optimized geometries and energies. This work provides a general understanding of the strengths of different small molecule force fields and identifies areas of improvement for future force field development.

## Methods

### We acquired reference geometries and energies of molecules from QCArchive and grouped them by connectivity

We obtained the molecule set in this work from QCArchive ^69^ from the dataset labeled OpenFF Full Optimization Benchmark 1 (accessed November 11, 2019), which was created for the purpose of benchmarking OpenFF-1.0 ^70, 71^. An initial preprint of this work was posted after benchmarking OpenFF-1.0, but subsequently we were able to include OPLS3e results and added benchmarking of OpenFF-1.1 and 1.2. It is important to note, then, that this dataset was not curated to present any force field in a particular light; it was selected for benchmarking OpenFF-1.0 and has been retained as-is for the present comparison. However, OpenFF-1.2 marked a substantial refit and used an expanded training set of molecules, selection of which was at least partially informed by benchmarking of OpenFF-1.0 ^72^. These training set changes meant we had to remove some structures from our benchmark set to ensure there was no overlap between training and test sets. Particularly, we removed 2398 structures from 419 molecules which were used for training the more recent OpenFF-1.2.

Overall, the benchmark set was chosen to include a broad range of drug-like compounds ^71, 73^. This QCArchive dataset contains QM geometry-optimized structures and energies at the B3LYP-D3BJ/DZVP level of theory ^74– 78^. This method and basis set were chosen by the Open Force Field initiative to provide reasonably accurate conformational energies and geometries at moderate computational cost ^52, 53^.

**Figure 1.**
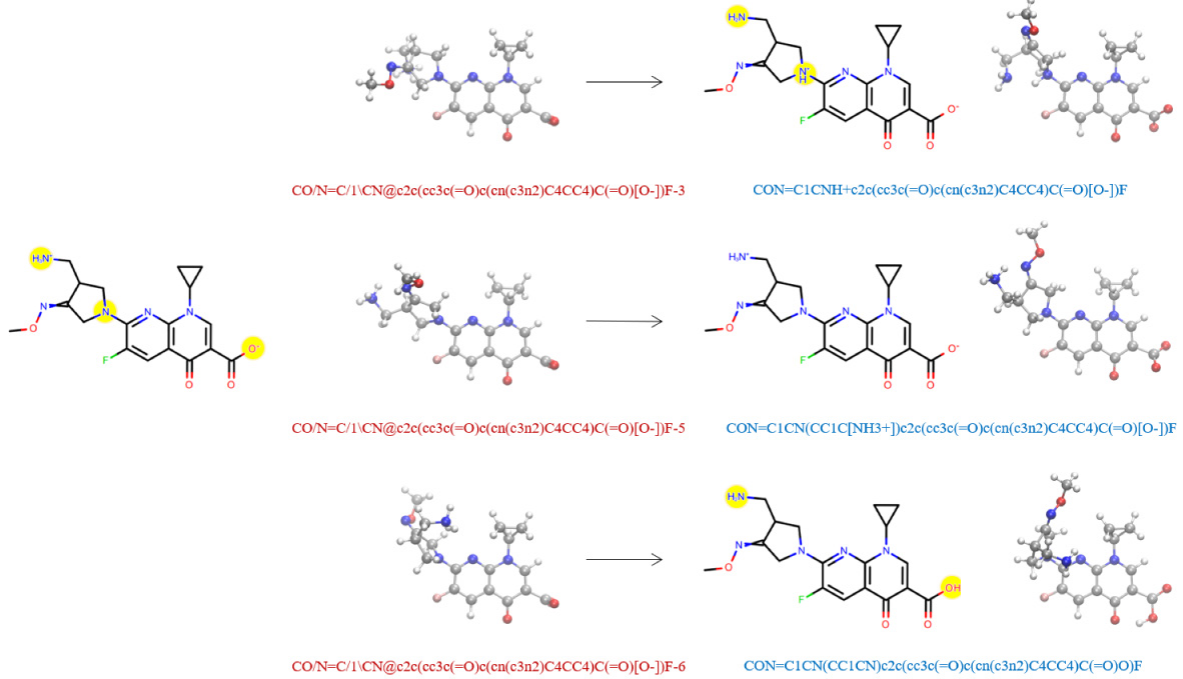
Molecules may change connectivity during QM optimization leading to different tautomers. On the left hand side, we show the Lewis structure and three associated conformers of an example molecule from the QCArchive OpenFF Full Optimization Benchmark 1 dataset. Yellow circles highlight the regions of potential tautomerization changes. The QCArchive SMILES labels are colored in red. The right hand side shows the structures after QM optimization. The canonical isomeric SMILES labels representing the optimized molecules are colored in blue. Only the middle structure retains the original tautomeric identity. In our dataset, the geometries on the right hand side would be analyzed as distinct molecules.

In our dataset, we organized molecular structures such that conformers of the same molecule were grouped together if they have the same absolute (non-isomeric) graph. Importantly, we do not use the SMILES string listed in the QCArchive DataFrame to represent the molecule itself, because the identity of the molecule may change during QM geometry optimization due to changes in bonding/tautomerization, such as shown in Figure 1. Molecules with different tautomerization states, which have different chemical connectivity, are treated as distinct molecules in our study. While two molecular structures may start QM optimizations from the same connectivity, we only use their final geometries to identify and distinguish molecules based on their connectivity. We grouped together all structures in the dataset whose final geometries yielded the same canonical isomeric SMILES string, as evaluated by OEMolToSmiles from the OpenEye OEChem Python toolkit ^79^. The structures were then organized into conformer sets as perceived by OEChem’s OEAbsCanonicalConfTest. This dataset organization procedure takes into account any molecular identity changes during QM optimization, such as if two molecules no longer had the same tautomerization state after QM optimization or if two different molecules ended up in the same tautomerization state. We ensured that what we identified as a molecule, and all of its given conformers, contained the same chemical connectivity.

The resulting QM geometries were used as input structures for gas phase energy minimizations using the following small molecule force fields: GAFF ^58^, GAFF2 ^59^, MMFF94 ^60– 63^, MMFF4S ^56, 65^, OPLS3e ^66^, SMIRNOFF99Frosst ^67^, and Parsley ^68^. The SMIRNOFF99Frosst version used here is SMIRNOFF99Frosst-1.1.0.offxml. SMIRNOFF99Frosst is a SMIRKS Native Open Force Field (SMIRNOFF) and descends from the AMBER parm99 force field as well as Merck-Frosst’s parm@frosst. Its successor is the OpenFF Parsley force field, for which three versions (1.0, 1.1, and 1.2) were evaluated, specifically openff_unconstrained-1.0.0-RC2.offxml (OpenFF-1.0), openff_unconstrained-1.1.1.offxml (OpenFF-1.1) and openff_unconstrained-1.2.0.offxml (OpenFF-1.2).

### We assigned FF parameters then energy minimized all molecules

Each structure was assigned AM1 Mulliken-type partial charges with bond-charge corrections (AM1-BCC charges) ^80, 81^ from the electrostatically least-interacting functional group technique. The partial charges were generated using the openmoltools wrapper ^82^ to OpenEye’s oequacpac charging engine ^79^ calling OEAM1BCCELF10Charges.

To assign force field parameters to each molecule, we used antechamber and tleap ^59^ via openmoltools ^82^ for the GAFF2 force fields. Parameter assignment as well as energy minimization for the MMFF94S force fields were handled using OpenEye oeszybki ^79^. The custom OPLS3e charge and parameter assignment was performed in two steps using Schrodinger Maestro (v. 2020-1 ^83^). First, ligprep ^84^ was used to convert ligands to Maestro format with settings to avoid modifying protonation or tautomeric states. Then ffbuilder was used to check for missing parameters and launch torsional drives with constrained minimization at the B3LYP/6-31G* level followed by single-point M06-2X/cc-pVTZ(-f) calculations. New OPLS3e parameters were derived for 1096 dihedrals, at a computational cost of about 100 CPU cores for 2 weeks to run high level DFT torsion fitting. This made the OPLS3e calculations substantially more costly; this may be in part because some of the benchmark set originates from eMolecules and consists of rather diverse and in some cases unusual chemistry which is not well captured by OPLS3e without additional parameterization.

We used the Open Force Field toolkit for SMIRNOFF99Frosst and Parsley, in all cases applying pre-assigned charges as described above. For the minimizations with OPLS3e, Schrodinger’s macromodel was used with the PRCG algorithm with a gradient tolerance of 0.05 kcal/mol. All other energy minimizations were completed in OpenMM ^85^ using the LBFGS algorithm with an energy tolerance of 5.0e-9 kJ/mol and 1500 maximum number of iterations.

We removed any molecular structure that was not successfully parameterized with all force fields. This set consisted of 721 structures that were unable to be parameterized by GAFF or GAFF2, 522 structures that raised an error during OpenMM setup through the Open Force Field toolkit, and 50 which had various OpenEye charging or stereochemical perception errors. Our pruned set going into energy minimization contained 22,675 structures from 3,271 molecules with unique chemical connectivity. Corresponding files containing QM geometries and energies, SMILES strings and depictions are deposited on GitHub, benchmarkff/molecules/set_v03_non_redundant/ ^86^. The repository also contains the structures removed due to parameterization or setup errors (in the benchmarkff/molecules/issues directory) and the structures removed due to overlap with the OpenFF-1.2 training set (in the benchmarkff/molecules/set_overlapping directory).

### We evaluated relative energies and geometric agreement with optimized QM geometries

We compared the energy-minimized geometries and energies for each force field with respect to the QM reference data by computing the following metrics: relative energy difference (ddE), root-mean-square deviation of atomic positions (RMSD), and torsion fingerprint deviation (TFD) ^87– 89^. The relative energy difference (ddE) between the FF and QM energy for the *i*th conformer of a specific molecule was computed using the following expression:

$$ddE_{i}=dE_{FF,\;i}-dE_{QM,\;i}\;(1)$$

=[FFenergy(i)−FFenergy(0)]−[QMenergy(i)−QMenergy(0)],(2)

where the 0th conformer is defined as the conformer with the lowest QM energy for the given molecule.

Molecules may change conformation after energy minimization, which may lead to lower agreement between FF and QM energies for minimizations beginning from a particular conformer. To address this potential issue, we performed a conformer matching process for each FF structure which considered the final optimized geometries and energy differences. We ensured that every MM conformer was within 1.0 Å RMSD of a QM reference structure. The QM reference conformer was removed from analysis if there were no FF conformers that matched it within 1.0 Å RMSD. Furthermore, if a molecule ends up with two of the “same” FF-minimized conformers compared to a QM reference structure, we only keep the FF conformer with the lowest RMSD score while any redundant conformers are removed from analysis. For this reason, the number of total molecular structures for each force field will likely differ after conformer matching as the intricate conformational energy landscapes are represented differently by various QM methods and force fields. Then, the mean signed deviation (MSD) was computed over all *N* conformers of each molecule with Equation 3, iterating over the relative energy *dE* of each conformer *i*. The reference conformer with *dE* = 0 was removed from the MSD calculation. The molecule MSDs were then represented in violin plots to compare among all force fields.

$$MSD=\frac{1}{N-1}\sum_{i=1}^{N-1}dE_{FF,\;i}-dE_{QM,\;i}\;(3)$$

To compare FF geometries with QM geometries, we used RMSD and TFD scores. The RMSD values, calculated with OpenEye OERMSD, took into account hydrogen atoms, symmetry-related transformations, and overlaid structures to yield the lowest possible RMSD. TFDs were computed using the RDKit Python library. We evaluated each of these three metrics individually and looked for potential correlations between energies and structures in terms of agreement with reference QM data.

We identified specific OpenFF-1.2 parameters which were overrepresented in high TFD regions. First, we collected all molecules having TFD scores above a visually determined cutoff of 0.12. We considered only molecules with distinct chemical connectivity. For each molecule in the high TFD group, we took the unique set of all parameters applied to the molecule. Thus, while a parameter may be applied multiple times to a single molecule, it would count only one time for that molecule. A parameter may be included multiple times when considering the entire TFD subset if it is applied to more than one molecule in the subset. For each parameter *i* in the force field, we computed its representation ratio as the fraction of molecules which apply that parameter:

$$\text{representation}\;\text{ratio}\;(i)=\frac{\text{nnumber}\;\text{of}\;\text{molecules}\;\text{which}\;\text{apply}\;\text{parameter}\;i\;\text{at}\;\text{least}\;\text{once}}{\text{total}\;\text{number}\;\text{of}\;\text{molecules}\;\text{in}\;\text{set}}\;(4)$$

This ratio was calculated for the high TFD subset as well as for the full set of molecules. To identify whether a parameter would be found more likely in the high TFD subset than the full molecule set, we compared the two representation ratios between the subset and full set using the one-sample Z-test for proportions. The population proportion for some parameter was designated as its representation ratio in the full molecule set, and the sample proportion was assigned to be the parameter’s representation ratio in the high TFD subset. We took the 95% confidence intervals from this Z-test to be the error bars for the representation ratios of the high TFD subset. Parameters having 20 or fewer molecules in the high TFD subset were excluded from further analysis and plotting due to inconclusive results from small sample sizes.

The complete Python code used for the setup, FF minimizations, and analysis of this work is open sourced and available on Github at https://github.com/MobleyLab/benchmarkff ^90^. An earlier version of this article can be found on chemRxiv (doi: https://doi.org/10. 26434/chemrxiv.12551867.v2). 

## Results and Discussion

Here, we present and discuss our results comparing several general small molecule force fields against reference QM data. We are interested in two major categories of comparison – energetic agreement and geometric agreement. Particularly, an ideal force field will yield the same energy minima or optimized geometries as the QM energy landscape, with no additional minima, and the relative energies of those minima will agree between QM and MM. Thus, to assess performance in these two categories, we computed relative conformer energies and compared these between MM and QM, as well as assessed geometric agreement of MM optimized geometries with those from QM. We also identified specific parameters for the improvement of future versions of the OpenFF small molecule force field.

Our study relies on the assumption that force field accuracy can be evaluated using gas phase energies and geometries. One of the greater goals of force field science, such as that of the Open Force Field Initiative, is building force fields that will work well in the condensed phase (e.g., small molecules in solution or binding to biomolecules). That being said, we make our assumption based on two key observations. First, force fields—especially those in the AMBER family—are usually fitted to reproduce gas phase conformational energies and geometries ^58^. This means that we are testing these force fields on properties they are fitted to reproduce. Second, bonded parameters are not expected to change significantly on transfer to the condensed phase. Rather, non-bonded interactions are particularly important in condensed phase simulations. Of the non-bonded interactions, electrostatics models are often polarized beyond what would be expected in the gas phase in order to reproduce condensed-phase properties, and Lennard-Jones parameters can be tuned to reproduce condensed phase properties (as has been a particular focus of the OPLS force fields ^91, 92^). Even when these are done, force fields retain bonded terms parameterized to reproduce QM geometries and energetics, further emphasizing the importance of testing in such a context. We therefore believe our assumption is reasonable and that this work warrants investigation.

We start our force field benchmark analysis by comparing FF energies to QM energies. Here, since our choice of reference energy for MM is arbitrary, we choose to compare relative conformer energies. For any given molecule, an ideal force field would have relative energies for different conformers in MM that agree with those for the same conformers in QM. For the differences in relative conformer energies that we computed—that is, the difference between the MM relative conformer energies and the QM relative conformer energies—a FF with greater agreement to QM should have more values around or at 0 kcal/mol, and a FF with lower agreement with QM would exhibit a broader distribution of values that are further away from 0 kcal/mol.

**Figure 2.**
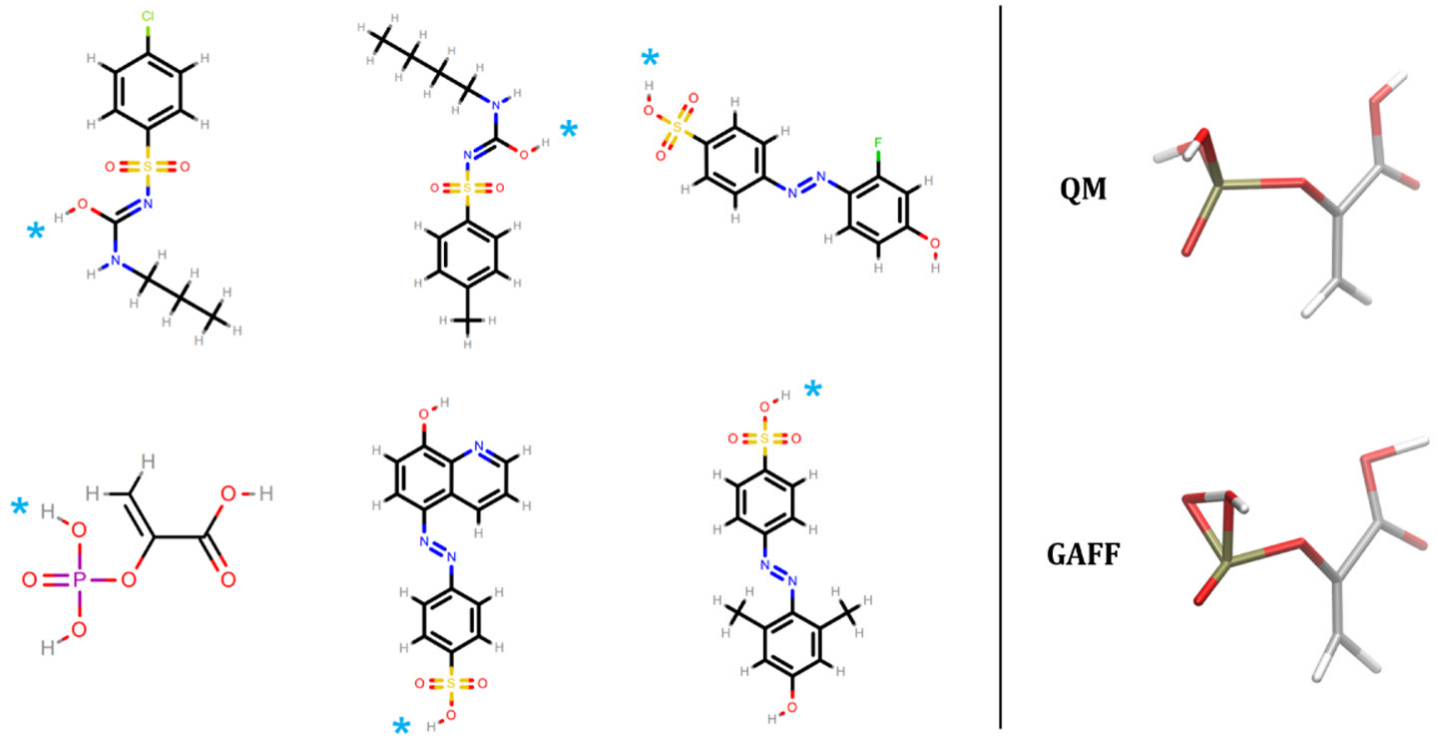
Molecules with extreme relative conformer energies for GAFF. The right hand side depicts the QM and FF geometries for phosphoenolpyruvic acid. The GAFF structure shows a representative overlap of a polar hydrogen atom with its connected parent atom due to a missing van der Waals parameter. On the left hand side, the overlapping hydrogen for the six molecules are denoted by cyan asterisks.

The relative conformer energies of all molecular structures in our dataset with the nine force fields were generally within ±50 kcal/mol of the energies of the most favorable QM conformers ( Table 1), and 95% of the relative conformer energies were within 11 kcal/mol. However, GAFF had outlying energies that were several orders of magnitude beyond this range (row 1 of Table 1). These energies were traced back to six molecules (62 conformers thereof) shown in Figure 2. These molecules all contain a polar hydrogen atom which, after geometry optimization, overlaps with its parent atom. The spurious overlap of these hydrogen atoms, and associated energy extremes, is due to a missing van der Waals parameter in GAFF. In GAFF2 (and SMIRNOFF99Frosst and subsequent OpenFF force fields ^11, 67, 68^), hydroxyl hydrogens no longer have zero Lennard-Jones parameters, which seems to eliminate the problem for these molecules. Similar collapse of hydroxyl groups in close proximity has been observed previously in force fields with zero LJ parameters for hydroxyl hydrogens ^11^.

**Table 1.** Minimum and maximum ddE values as computed in Equation 2 for all structures of each force field. Energy units are in kcal/mol.

Force field	min ddE	max ddE
GAFF	-35002325.4	5549.7
GAFF ^a^	-44.1	14.8
GAFF2	-43.7	15.6
MMFF94	-52.1	29.8
MMFF94S	-49.5	25.1
SMIRNOFF99Frosst	-42.8	18.8
OpenFF-1.0	-38.4	19.3
OpenFF-1.1	-38.6	18.3
OpenFF-1.2	-37.9	15.4
OPLS3e	-30.4	9.6

^a^ With outliers removed

**Figure 3.**
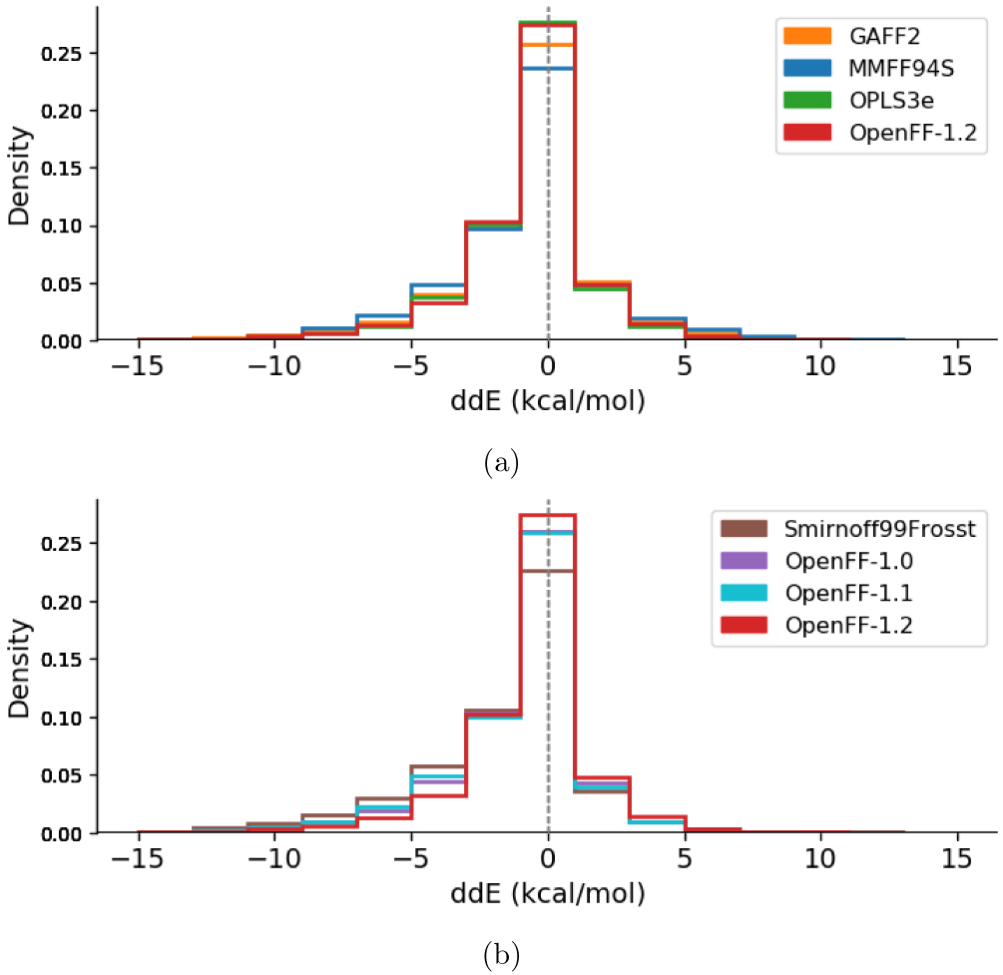
Histograms of the relative conformer energy differences as computed in Equation 2 for each force field relative to QM. Each molecular structure, including different conformers of the same molecule, is counted separately. Since the global minimum molecular structures were set to zero deliberately and add a constant offset to the central bin, they are removed from the counts. A force field having higher agreement with QM would have a higher bin centered at ddE = 0 kcal/mol. ( **a**) compares the latest release of all four force field families. ( **b**) shows the four histograms belonging to the OpenFF family of force fields. OpenFF-1.0 (purple) and OpenFF-1.1 (light blue) overlap in the central bin. The corresponding graph comparing histograms of all calculated force fields can be found in the *Extended data*, Figure S.1 ^93^.

After excluding the 62 GAFF outliers, the ddE energies are histogrammed in Figure 3 and *Extended data*, Figure S.1 ^93^. The difference between MM relative conformer energies and QM relative conformer energies exhibit very similar distributions for all force fields. All distributions appear asymmetric, having a skew towards more negative ddE values than positive ones, indicating that the conformer energy differences may be underpredicted by MM compared to QM. Force fields of the same family tend to be more consistent with each other (GAFF and GAFF2, MMFF94 and MMFF94S), see *Extended data*, Figure S.1 ^93^. From these results, the qualitative ordering of force fields from lowest to highest agreement with QM energies goes as SMIRNOFF99Frosst < MMFF94 ∼ MMFF94S < GAFF ∼ GAFF2 ∼ OpenFF-1.0 ∼ OpenFF-1.1 < OpenFF-1.2 < OPLS3e. In other words, the peak size around ddE = 0 kcal/mol (the fraction of molecules described particularly well) is greatest for OPLS3e, closely followed by OpenFF-1.2. OPLS3e predicts 55.3 ± 0.3% of conformers within 1 kcal/mol of QM, with OpenFF-1.2, GAFF2, and MMFF94S identifying 54.8 ± 0.3%, 51.3 ± 0.3%, and 47.0 ± 0.3% respectively. By this metric, OPLS3e and OpenFF-1.2 seem to exhibit roughly similar performance, with the other force fields performing somewhat worse. Figure 3b illustrates the progress made within the OpenFF family of force fields. The predecessor SMIRNOFF99Frosst performs worst of all investigated force fields and is improved upon by the first releases OpenFF-1.0 and OpenFF-1.1, which show intermediate performance. Finally, the most recent release OpenFF-1.2 indicates further improvement.

**Figure 4.**
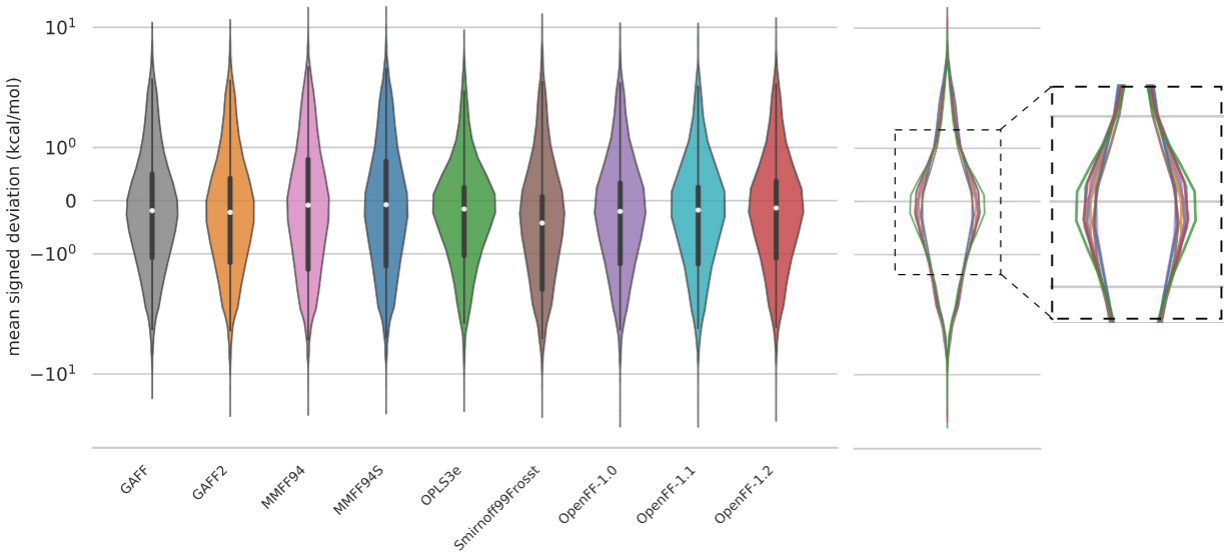
Violin plots of the mean signed deviations of MM conformer energies relative to QM conformer energies as computed in Equation 3. The energy MSDs only take into account structures matched within 1 Å of the QM reference structure, so there are minor differences in the amount of data used to plot each violin (see text). To correct for this, each plot was scaled to the same area. The vertical axis is shown on a logarithmic scale. An overlay of the violin plots on the right panel better shows the subtle distinctions between the force fields in the most populated region, near zero error. An equivalent graph with an RMSD threshold of 0.3 Å is shown in the *Extended data*, Figure S.2 ^93^.

Given that two conformers starting from the same geometry may optimize to two distinct conformers after FF minimization, we took another approach to analyzing energy distributions, only considering the FF conformers that correspond to a QM counterpart. A FF conformer is deemed to have a “match” with a QM conformer if its RMSD is less than or equal to 1 Å (see more details in Methods). The number of matched conformers for each force field are: 20,815 (GAFF), 20,836 (GAFF2), 20,674 (MMFF94), 20,684 (MMFF94S), 21,961 (OPLS3e), 16,177 (SMIRNOFF99Frosst), 19,103 (OpenFF-1.0), 17,965 (OpenFF-1.1), 21,428 (OpenFF-1.2). The mean signed deviation of the matched conformer energies are shown as violin plots in Figure 4. The violin plots are scaled such that each violin has the same area. This figure shows that the mean signed deviation of relative conformer energies is also fairly consistent between different force fields as seen in Figure 3. Upon closer inspection, the violins for OPLS3e and OpenFF-1.2 are slightly wider around 0 kcal/mol (and narrower elsewhere), signifying marginally higher agreement with QM energies. Equivalent results for an RMSD threshold of 0.3 Å to the QM structure is shown in the *Extended data*, Figure S.2 ^93^. With this lower RMSD criteria, the number of structures within the cutoff is roughly halved compared to a threshold of 1 Å while the ranking of force fields remains unaltered. Note that this conformer filtering step was only used for analyzing the energies in the violin plots, and other results throughout this work do not rely on matched conformers.

**Figure 5.**
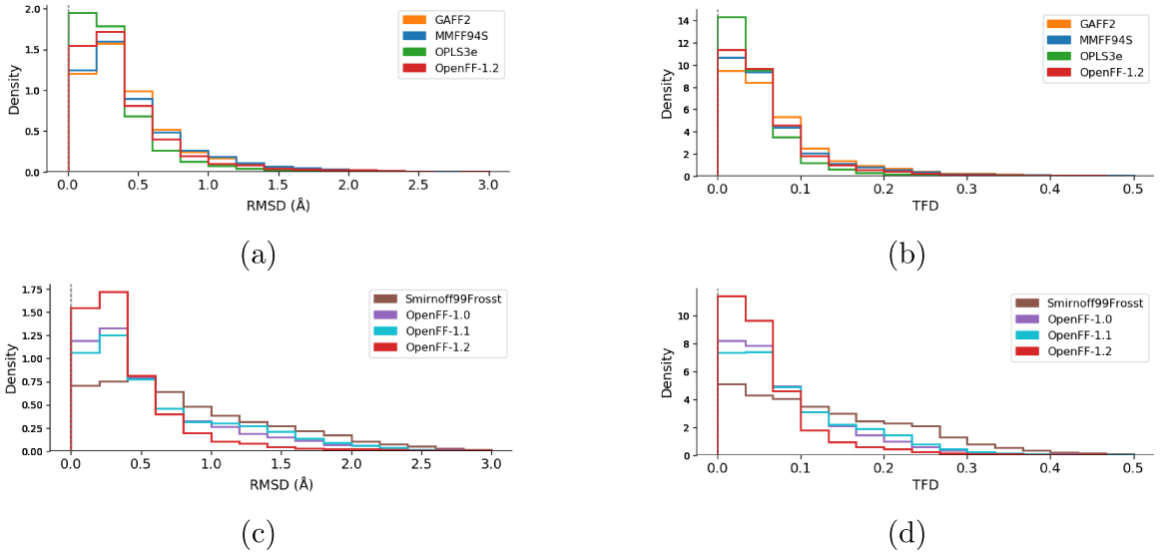
Histograms of the RMSD ( **a, c**) and TFD ( **b, d**) values between force field structures as compared to QM structures. Values closer to zero indicate higher geometric similarity for both RMSD and TFD. Panels ( **a**) and ( **b**) compare the families of force fields (GAFF2, MMFF94s, OPLS3e, and OpenFF-1.2). Panels ( **c**) and ( **d**) compare the force fields of the OpenFF family (Smirnoff99Frosst, OpenFF-1.0, OpenFF-1.1, and OpenFF-1.2). The corresponding graphs with histograms of all force fields are shown in the *Extended data*, Figure S.3 ^93^.

We next examine agreement between FF-optimized geometries and those from QM, as calculated by each molecule’s RMSD and TFD scores with reference to the parent QM-optimized geometries. While RMSD is the more common metric, it may depend on the molecule size, complicating interpretation of geometric agreement ^94, 95^. In contrast, TFD was designed to be more independent of molecule size in order to compare molecular conformations more meaningfully ^87^. This can help offset issues with RMSD where larger, more ﬂexible molecules can contribute the most to RMSD. The TFD score between two molecular structures is evaluated by computing, normalizing, and Gaussian weighting the (pseudo)torsion deviation for each bond and ring system. While TFD is normalized from 0 to 1, RMSD is unbounded. Both RMSD and TFD are similar in that a higher value signifies lower agreement between the geometries of two molecules. A FF which yields optimized geometries closer to those of QM would have generally smaller RMSD/TFD values. We calculated RMSD and TFD scores for all MM optimized geometries with respect to QM geometries. We plotted this data in histograms in Figure 5.

In terms of geometry agreement, we observed similar results between the RMSD and TFD plots. The ranking of the force fields is mostly the same as with the *ddE* rankings above, with OPLS3e performing best followed by the latest open force field release, OpenFF-1.2. One major difference is the ranking of MMFF94S over GAFF2, while the latter had better agreement with QM in terms of *ddE*. The OpenFF force fields show clear improvement with newer versions by having higher densities close to zero and also by having tails successively reduced. Although SMIRNOFF99Frosst had a non-negligible density at RMSD > 2 Å, virtually all structures optimized with OpenFF-1.2 agree with the QM structures with RMSD < 2 Å ( Figure 5c). Both TFD and RMSD distributions show qualitatively the same ranking of force fields, whereas the quantitative differences appear to be of different magnitudes. For example, MMFF94S is very close to GAFF2 in terms of RMSD ( Figure 5a). According to TFD, MMFF94S appears to be closer to OpenFF-1.2, with GAFF2 having less agreement with QM ( Figure 5b).

**Figure 6.**
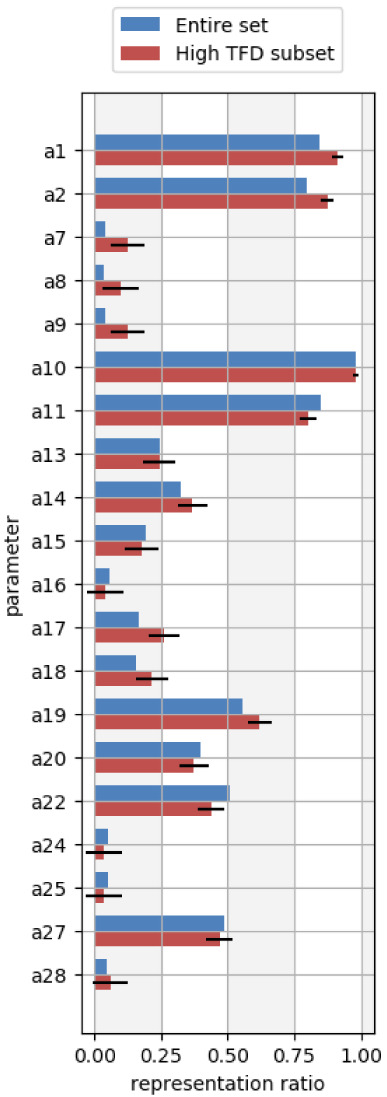
Prevalence of a subset of angle parameters used in the OpenFF-1.2 force field as calculated by Equation 4. The blue bars represent the parameter ratios from the full molecule set, and the red bars represent the parameter ratios from only the set of molecules with TFD values greater than 0.12. Error bars denote the 95% confidence interval determined from the one-sample Z-test for proportions. Parameters which are estimated to be overrepresented in molecules with high TFDs have statistically significant differences between the full set and high TFD set of parameter ratios (also see Table 2). Parameters with statistically significant differences in this plot are a1, a2, a7, a8, a9, a17, a18, and a19.

From the histograms, we can identify areas for force field refinement of molecular geometries by analyzing molecules with significant conformational differences from the QM reference (molecules with TFD values > 0.12), and in particular by focusing on parameters which occur more frequently than expected in such molecules. Parameters which are overrepresented in molecules with significant deviations are more likely to be responsible for such deviations. To assess this, we computed the representation ratio ( Equation 4) for each OpenFF-1.2 force field parameter in both the high TFD molecule subset as well as in the full set of molecules. We estimated whether each parameter was applied more frequently in the high TFD subset compared to the full set by computing the one-sample Z-test for proportions. Figure 6 shows the results for a subset of the OpenFF-1.2 force field parameters, wherein the parameters of interest have a statistically significantly higher representation ratio in the high TFD subset within a 95% confidence interval. These parameters are listed in Table 2 for the complete OpenFF-1.2 force field, and likely warrant further investigation as a possible cause of deviations from the QM reference. The complete set of OpenFF-1.2 representation ratio plots are placed in the *Extended data*, Figures S.4-S.6 ^93^.

**Table 2.** OpenFF-1.2 force field parameters identified to be overrepresented in high TFD molecules. These parameters show statistically significant differences (p < 0.05) in representation ratios of the high TFD molecules compared to ratios of the full molecule set. Refinement of these parameters may address conformational differences in MM-optimized molecular geometries compared to QM-optimized geometries.

Angles	Bonds	Improper dihedrals	Van der Waals	Proper dihedrals
a1	b1	i3	n2	t1
a2	b2		n3	t2
a7	b3		n16	t3
a8	b7		n20	t4
a9	b9			t17
a17	b10			t18
a18	b83			t20
a19				t22
				t23
				t51
				t52
				t59
				t61
				t62
				t68

**Figure 7.**
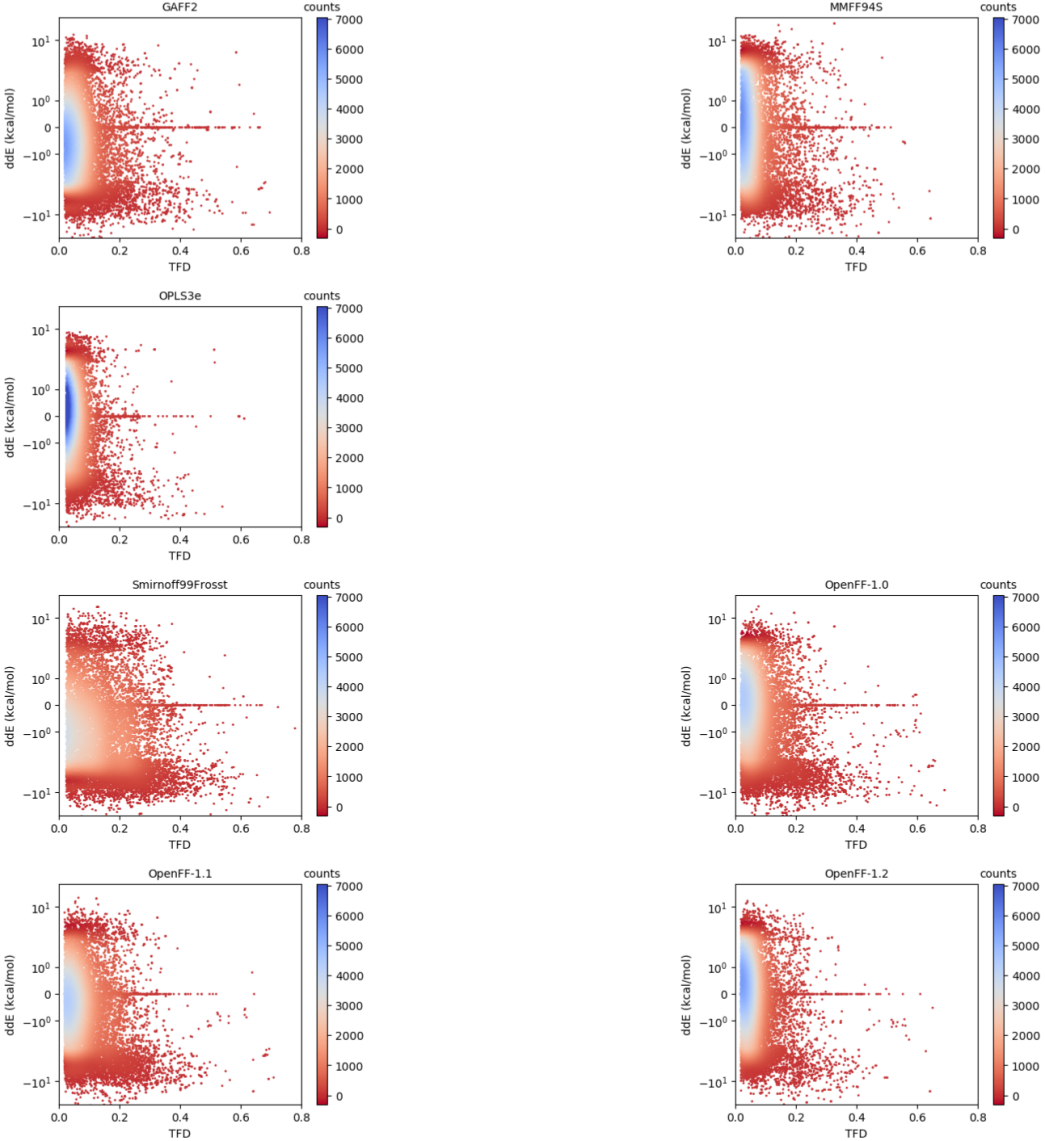
Scatter plots of relative conformer energies (ddE) versus TFD scores. The points are colored by the interpolated density of points in a certain area. Blue indicates region of high density, that is, high compactness of points in that area. A force field having better agreement in both relative energies and geometries with the QM reference would have more points around the origin (ddE = 0, TFD = 0), though it is presumably possible for a force field to improve along one axis without improving along the other. The vertical axis is represented on logarithmic scale; the same plots with linear scaling can be found in the *Extended data*, Figure S.7 ^93^.

We then sought to determine if there was a dependence between the relative energies and geometries. Scatter plots of ddE versus RMSD/TFD are shown for all force fields in Figure 7. Each structure in our dataset is plotted as a single point. The ddE values are plotted on a logarithmic scale. We include in the *Extended data*, Figure S.7 analogous plots with ddE represented on a linear scale ^93^. Given tens of thousands of points on each plot leading to many overlapping points, we applied a color gradient from red to blue to represent regions from low to high density, respectively. Similar to the data represented as one-dimensional histograms ( Figure 3 and Figure 5), a higher density of points at the origin indicates results in better agreement with the reference QM data. There seems to be no general correlation between the energies and geometries. However, using this visualization we identified particular chemical moieties that represent outlying energies or geometries (vide infra).

**Figure 8.**
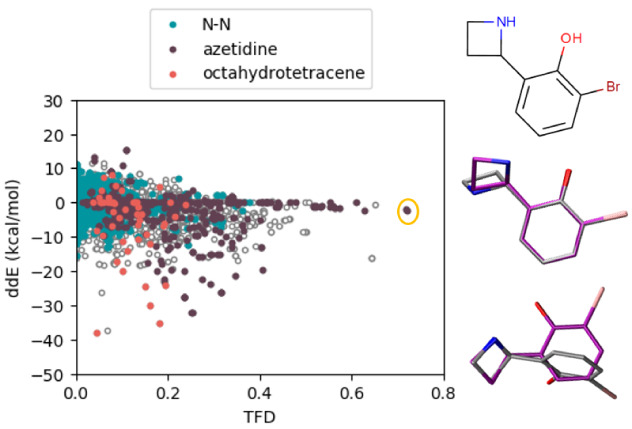
Scatter plot of the OpenFF-1.2 force field of relative conformer energies versus TFD scores. Colors highlight particular chemical groups that appear to be systematic outliers in energies or geometries. On the right hand side, we show a figure with high TFD and low ddE as circled in the scatter plot. The QM structure is in purple, and the force field structure is colored in silver. Analogous plots for all other force fields are shown in the *Extended data*, Figure S.9 ^93^.

In this diverse set of molecules, we point out three particular moieties, those containing an N-N single bond (3824 structures), those containing an azetidine ring (543 structures), and a highly substituted octahydrotetracene (50 structures). These subsets are highlighted for OpenFF-1.2 in Figure 8 (see *Extended data*, S.9 for other force field results ^93^). Molecules containing an N-N single bond have a wide spread of energies with several ddE outliers between -10 to -20 kcal/mol. Structures with azetidine revealed both energy and geometry outliers in the Amber and OpenFF force field families. Lastly, the substituted octahydrotetracene scaffold was found to be challenging to all force fields in reproducing QM energies (an example is presented in the *Extended data*, Figure S.8 ^93^). These moieties represent systematic outliers that can be used in future studies investigating particular shortcomings of force fields or improving future versions of force fields. Indeed, some of these issues have been a focus of fitting of the OpenFF 1.1 and 1.2 force fields ^96^. We have calculated the average and standard deviation statistics of ddE and TFD for the whole set of structures and the subsets containing these moieties. The results are listed as *Extended data*, Table 1 and Figure S.10 ^93^. Both the spread and average of the distributions of the subset are generally larger than the ones of the whole set, emphasizing that these moieties are challenging to be parameterized. For the OpenFF family of force fields, a clear improvement in these statistics can be seen for the newer versions, especially for the N-N moiety (both TFD and ddE) and the octahydrotetracene (in terms of ddE).

## Conclusions

In this work, we presented a large-scale analysis of nine small molecule force fields in terms of their relative conformer energies and geometries compared to reputable QM data. Amongst the force fields (GAFF, GAFF2, MMFF94, MMFF94S, OPLS3e, SMIRNOFF99Frosst, OpenFF-1.0, OpenFF-1.1, and OpenFF-1.2), OPLS3e performed best in terms of reproducing QM conformer energies and geometries. However, it is worth noting the higher computational cost of the high level DFT torsion fitting for generating the optimal OPLS3e parameters (likely in part due to the diversity of the present molecule set), whereas with the other force fields this step was rapid.

The OpenFF versions showed improvements in both metrics with each new version, and the latest OpenFF-1.2 appears to be approaching the degree of accuracy of OPLS3e, at least on this dataset. This is despite the extra dihedral parameter fitting with OPLS3e. Thus OpenFF-1.2 seems to be positioned as the best open source/free small molecule force field in this study, as OPLS3e is proprietary.

Other aspects of interest included the ability of MMFF94 and MMFF94S to capture QM geometries better than several other force fields, but still not as well as OPLS3e or OpenFF-1.2, especially when using a more size-independent geometry measure. Finally, we identified particular chemical moieties that were systematic outliers in terms of relative energies or geometries. These N-N, azetidine, and octahydrotetracene-like compounds represent potential areas for improvement in future force field development.

Our work also highlights the progress the Open Force Field Initiative has made towards its goal of producing high quality public, open force fields built with infrastructure which enables rapid parameterization. Particularly, the series of OpenFF force fields presented here demonstrate marked improvements in accuracy over a relatively short time, and these improved force fields are available to everyone. One key challenge going forward will be to continue improving treatment of problematic areas of chemical space and expanding coverage. In parallel, future OpenFF updates will include improved treatment of torsions (via Wiberg bond order-based parameter interpolation ^97^ which was recently implemented in our toolkit) and better handling of trivalent nitrogen geometries ^98^ which we hope will boost performance further.

Beyond these specific conclusions, we believe the general strategies employed here for benchmarking force field performance will be useful far more broadly than this specific study. Particularly, comparing performance by both geometric and energetic measures is particularly important, as the analysis we have done demonstrates. Additionally, the availability of a large amount of public data in QCArchive facilitates straightforward large scale benchmarking in a way it has not been done previously.

We share our Python code comprising the setup, minimization, and analysis of this research on Github, available at: https://github.com/MobleyLab/benchmarkff ^90^.

## Data availability

### Underlying data

Zenodo: Molecular geometries and energies from quantum mechanical calculations and small molecule force field evaluations. https://dx.doi.org/10.5281/zenodo.4247859 ^99^.

### Extended data

Zenodo: Supporting Information: Molecular geometries and energies from quantum mechanical calculations and small molecule force field evaluations. http://dx.doi.org/10.5281/zenodo.4299200 ^93^

This project contains the following extended data:

- Histograms for all force fields regarding energies of conformers, RMSD and TFD relative to QM reference data for all force fields investigated in this work
- Plots similar to those in Figure 7 with linear scaling of the vertical axis
- Plots in the same manner of Figure 8 for all force fields in this work
- Average and standard deviation statistics of relative energies and TFDs for different (sub)sets of structures
- An example of one of the octahydrotetracene-based structures having high deviation in ddE

Data are available under the terms of the Creative Commons Attribution 4.0 International license (CC-BY 4.0). 

## Code availability

Source code used in conducting the modeling, analysis and plots is available on GitHub, with the specific version used here archived on Zenodo.

- Source code available from: https://github.com/mobleylab/benchmarkff
- Archived source code at time of publication: https://dx.doi.org/10.5281/zenodo.4252694 ^90^
- License: MIT
