# Supplementary material for: Benchmark assessment of molecular geometries and energies from small molecule force fields
Source: F1000Res. 2020 Dec 3;9:Chem Inf Sci-1390. [Version 1] doi: 10.12688/f1000research.27141.1 (PMC7863993; doi:10.12688/f1000research.27141.1)
Supplement: Peer review history

# Peer review: Reviewer response for version 1

**DOI:** 10.5256/f1000research.29983.r77769

**Published:** 2021-02-04

**Authors:** Sreyoshi Sur

**Affiliations:** 1 Moderna, Cambridge, MA, USA

In this paper, the authors have looked at how closely do the molecular mechanics and quantum mechanics optimized data match for small molecules in gas phase. In order to achieve this they used two parameters:

-  Conformer relative energies, calculated using different forcefields and the corresponding quantum mechanically calculated energies.
- Energy minimized geometric structures evaluated using the forcefields and the QM calculations. 

They provided a list of 3271 molecules with 22675 different structures. Molecules were selected based on unique chemical connectivity and whether all the molecules can be parametrized by GAFF, GAFF2, MMFF94, MMFF4S, OPLS3e, SMIRNOFF99Frosst, and OpenFF Parsley (1.1,1.2,1.3). Molecules that were part of training set for the forcefield OpenFF Parsley was removed for evaluation too. For every evaluated structure, first the authors compared each molecules conformers QM calculated and the MM energies. Their results show that for a greater number of molecules OPLS 3e evaluated energies were closest to the QM energies, followed by OpenFF 1.2.

  They also evaluated MM energy for the conformers which most closely matched the energy minimized QM structures. In this case also OPLS3e and OpenFF 1.2 performed better than the rest of the molecules. To compare the 3D structures, two metrics were used, RMSD and Torsion fingerprint deviation. Using these two metrics very slight changes were observed in terms of the relative energies and the two forcefields which match closest to QM remain the same (OPLS 3e and OpenFF 1.2).

 Comments: The paper addresses most of the concerns in terms of comparing small molecule's structure and energies w.r.t different MM forcefields. The authors have provided their GitHub repository consisting of all the test data and their code which was used to obtain the results. This enables the reader to reproduce the results of the paper and also have a workflow to run some of these QM vs MM comparisons with completely new sets of molecules. 

 Questions/Minor revisions for the authors:

1. For Figure 7, I would suggest rearranging the plots so that they are next to each other and thus easy to compare.
2. For Figure 7, the authors can also show a difference plot between OPLS3e and OpenFF 1.2 and between OPLS3e and MMFF945, then the subtle changes between OpenFF1.2 and MMFF945 around ddE=0 compared to OPLS3e will be more prominent.
3. For the 3271 molecules chosen, what is the overall chemical diversity of these molecules?
4. These small molecules are usually used as ligands to proteins, can the authors comment on how these improvements in the OpenFF 1.2 will impact binding affinity calculations?

Is the work clearly and accurately presented and does it cite the current literature?

Yes

If applicable, is the statistical analysis and its interpretation appropriate?

Yes

Are all the source data underlying the results available to ensure full reproducibility?

Yes

Is the study design appropriate and is the work technically sound?

Yes

Are the conclusions drawn adequately supported by the results?

Yes

Are sufficient details of methods and analysis provided to allow replication by others?

Yes

Reviewer Expertise:

Computational Chemistry and Computational Biophysics

I confirm that I have read this submission and believe that I have an appropriate level of expertise to confirm that it is of an acceptable scientific standard.

# Peer review: Reviewer response for version 1

**DOI:** 10.5256/f1000research.29983.r75766

**Published:** 2020-12-22

**Authors:** Sereina Riniker

**Affiliations:** 1 Laboratory of Physical Chemistry, Department of Chemistry & Applied Biosciences, ETH Zürich, Zürich, Switzerland

**Summary:**

 The authors describe the benchmarking of different general force fields for small organic molecules against QM reference data in terms of gas-phase geometries and energies. A large dataset of 3271 molecules with 22675 conformers was assembled and is freely available. Although the force fields are typically aimed for condensed-phase systems and the dataset provides gas-phase data, such a benchmarking set and the accompanying analysis is highly valuable for the force-field community. Python code is also available on Github. From the results, it is encouraging to see that systematic improvement of force fields is possible. Minor revisions are suggested below to further improve the clarity and quality of the article.

**Suggested Revisions:**

- Dataset: The authors had to remove the 419 molecules used for training of OpenFF-1.2 from the QCArchive dataset to ensure an unbiased test set. Did this lead to a certain enrichment or depletion of particular functional groups in the remaining test set?
- QM reference data: How much variation in the QM ddE would you expect for different functionals/basis sets? In other words, what “error” (or uncertainty) is associated with the QM calculations? An indication of the variation between different QM methods is given in Faraday Discuss., 195, 497-520 (2016).
- The authors compare the ddE between the QM and MD approaches. However, can we expect the deviations to be systematic?
- Table 1: It would be interesting to see the min and max ddE for GAFF after the removal of the six molecules with collapsing hydroxyl groups (maybe in parentheses after the original values?).
- Fig. 3 (and Fig. S1): I think there should be a sufficiently large number of data points to choose a smaller bin size for the histograms.
- Fig. 7: It appears that there is a straight line around ddE = 0, i.e. conformers with a large variety in TFD all have a ddE of zero. Is there an explanation for this?
- The TFD does not give equal weights to all torsions in a molecule: (1) all bonds in rings are combined to a single value in the torsion fingerprint, and (2) torsions in the centre of the molecule are weighted more than terminal torsion angles. In the calculation of ddE, on the other hand, all deviations contribute similarly to the energy difference. Could this be one of the explanations that there is basically no correlation between higher ddE values and higher TFD values? I would suggest to consider a torsional-angle RMSD as an alternative measure for the structural differences.
- How important is the targeted torsion fitting in OPLS3e for its performance? In other words, would you expect the performance of e.g. OpenFF-1.2 to improve significantly with custom torsions?

Is the work clearly and accurately presented and does it cite the current literature?

Yes

If applicable, is the statistical analysis and its interpretation appropriate?

Yes

Are all the source data underlying the results available to ensure full reproducibility?

Yes

Is the study design appropriate and is the work technically sound?

Yes

Are the conclusions drawn adequately supported by the results?

Yes

Are sufficient details of methods and analysis provided to allow replication by others?

Yes

Reviewer Expertise:

Computational chemistry

I confirm that I have read this submission and believe that I have an appropriate level of expertise to confirm that it is of an acceptable scientific standard.
